# CT-Scan sheep and human inner ear morphometric comparison

**DOI:** 10.1016/S1808-8694(15)30971-X

**Published:** 2015-10-19

**Authors:** Valter Ayres Seibel, Luiz Lavinsky, Klaus Irion

**Affiliations:** a PhD, MD; b PhD, MD; c PhD, MD. Universidade Federal de Rio Grande

**Keywords:** experimental surgery, animal models, sheep

## Abstract

**Introduction:**

Studies about the use of sheep in surgical training and experimental otologic surgery are rare. This study intends to contribute to the knowledge on this field.

**Aim:**

To study sheep inner ear structures using computerized tomography and serial cross-sections to collect more accurate morphometric data to compare sheep and human ears.

**Material and methods:**

This descriptive study compared the inner structures of sheep and human ears. Measurements were made using computerized tomography, and they were stored in a DICOM compact disc for later analysis and manipulation, with a program used for medical image analysis (Osíris 4.16).

**Results:**

Mean measures for sheep and human ears were found to be similar in this morphological study. Most structures (10 out of 15) maintained the 2/3 ratio of sheep to human ear.

**Conclusion:**

The results of this morphometric study of sheep ear are an important contribution to the development of an animal model to be used for surgical training and experimental otologic surgeries.

## INTRODUCTION

The scarce information we have about the use of sheep as an anatomical model for experimental surgery and otologic surgery training, come from the recent past and from investigations done by our local research group^1^, ^2^, ^3^, ^4^, ^5^, ^6^, ^7^, ^8^. This has motivated the author to design and execute the present study.

The growing need to enhance the otology surgeon's skills and the discovery and testing of new surgical approaches, as well as experimentation with new materials employed in this field are the reasons for this constant search for more available and alternative surgical models for experimentation, in order to aid the teaching-learning process^3^, ^6^. The more similar to the human being these models are, the better.

Today, the most used middle size animals for such experiments are dogs, cats and monkeys. These animals, besides presenting significant anatomical differences when compared to the human being, are more aggressive, more prone to developing diseases and are more demanding as to the maintenance and housing when compared to sheep.

Lavinsky e Goycoolea^2^ presented experiments in sheep. Besides being docile enough to be handled in the lab, these animals are more robust, less prone to diseases and do not require as much care as the animals aforementioned, and they may return to the farms after the otologic surgeries^2^, ^7^. Thus, their maintenance is much more economical. Another factor of real importance, according to these papers is the important anatomical similarities between their ear and the human ear^2^, ^3^, ^7^, ^8^, having an estimated 2/3 size ratio with the human ear^7^, ^8^.

For all the reasons aforementioned, the sheep is an excellent anatomical model option for otologic surgery training and experimentation. The present study aims at quantifying and broadening the little information existing, which are very descriptive. The sheep ear was studied through CT scan and successive 0.5mm slices in order to carry out a more precise morphometric study both in shape and measurements.

The goals of the present paper are:

• To morphometrically compare the main sheep inner ear structures, in other words the anterior and posterior labyrinths.
• Compare the anatomical structure dimensions of human and sheep inner ears, considering that sheep structures are equal to 2/3 of human structures in size ratio.
• Contribute to create a model for otologic training and experimental surgery.

## MATERIALS AND METHODS

At outlining, we carried out a descriptive study without follow up, in which we compared the inner ear structures of sheep with those from human beings.

### Materials

**Figure 1:**
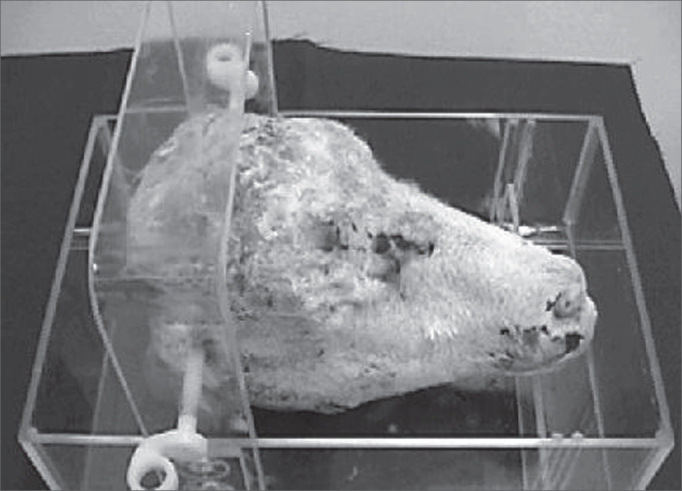
Acrylic support with the sheep head.

We used 19 heads from sheep which were slaughtered for human consumption and stored in a 220L freezer, corresponding to 38 temporal bones from Corriedalle with Texel sheep. A Toshiba CT scan machine, model Xvision EX and a support with acrylic fixator was used to keep the sheep head in a fixed position during the CT scans, created and developed by the author (Figure 1).

The control images were made up of CT scans from 7 patients, corresponding to 14 human temporal bones. The CT scans were carried out in the conventional fashion and the measurements followed the same techniques used for the sheep CT scans.

### Head storage

The anatomical specimens were kept at -25°C until the CT scans were made.

The sheep head was placed in the proper acrylic support, where two plastic screws pinned it by the external acoustic meatus, and the mandible rested on a support, in order to undergo CT scan.

The sheep ears CT scan were carried out by an experienced radiologist.

The CT scan study was carried out with a spiral CT scanner, Toshiba, Xvision EX model machine, with a second turn time within the tube. The exams were done according to the following protocol:

1) Guidance scanning obtaining a digital radiogram in profile in order to plan the CT scan slices;
2) Simple axial slice, at the external auditory canal plane, to check for proper positioning;
3) Additional simple axial slices, until the position of the anatomical specimen was considered satisfactory in terms of is inclination and rotation;
4) Spiral scan with X-Ray beams collimation in 1mm, small sectional area, equivalent to 240mm, complete turn time in 1 second, table shifting speed defined to be 1mm per second (pitch 1), 120 kV, 50 mA, convolution filter for bone algorithm (FC30 - Toshiba) at a total scan time of 50 seconds or less.

The scanning duration varied according to the sizes of each anatomical specimen, according to the need for the scanning to cover all the petrous bone extension from its posterior border, as it was assessed by the planning digital radiogram. The data volume acquired during spiral scanning was then presented through contiguous axial images at every 0.5mm, originally in the coronal plane, with vision area focused in each ear, for the highest magnification possible, depending on the size of each petrous bone. In a total, we had at lease 37 direct axial images including both ears from each sheep in a single field of vision (reconstruction diameter), which varied between 70.31mm and 120.00mm, with pixel size varying between 0.137 × 0.137mm and 0.234 × 0.234mm. These ultra thin slices, acquired with 1mm collimation and reconstructed at every 1mm were planned in order to yield the best resolution possible and thus, discriminate the largest possible number of minute structures present in the sheep ears. The images, at every 0.5mm, were planned to have the best quality and reconstruction resolution in multiple planes (multiplan reconstruction). The acquisition matrix was selected at 512 × 512 pixels, with 16 bits of gray scale.

Each one of the anatomical specimens was positioned in a fixation support (Figure 1) with plastic pins inserted into the external auditory canal and with an adjustable support to elevate the mandible, until all axial slices presented the same coronal plane, for each one of the specimens. The variation in pixel size and reconstruction diameter aimed at obtaining the largest magnification where two ears could fit. Thus, pixel size varied between 0.137 × 0.137mm and 0.234 × 0.234mm; and the reconstruction diameter varied between 70.31mm and 120.00mm.

### Measurements

The CT scans were stored in a Dicom standard CD-ROM for later analysis and use with specific software for the analysis of medical images called Osiris 4.16. In this software, the images were assessed as to the sizes of certain structures, using the multiplan reconstruction resources, which allowed us to obtain measurements in the three planes: sagittal, coronal and axial. Multiplan reconstructions also facilitated the identification of anatomical points of reference which were used as reference points for each one of the structures measured. These were later plotted and analyzed through Microsoft Excel electronic spreadsheet.

**Figure 2:**
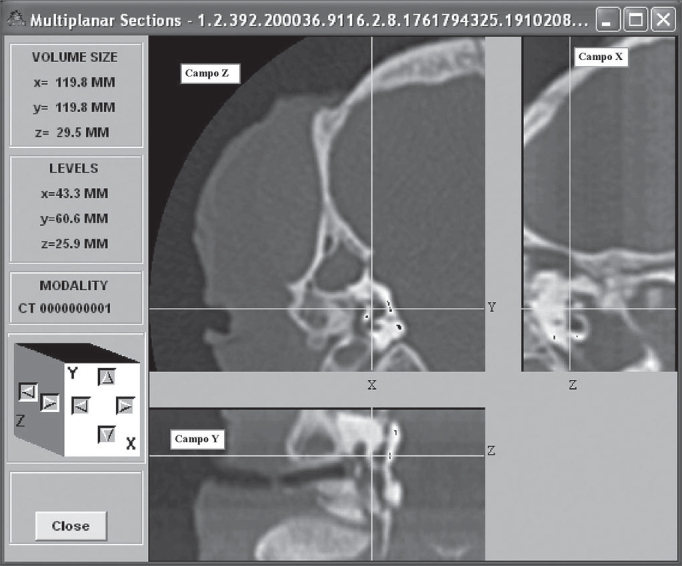
Image model in the Osiris 4.16 software.

Measurements were obtained by placing the cursor in each one of the structure borders, oriented in the coronal, axial or saggital planes (Figure 2).

In order to better understand the multiplan images, the coronal slice was called field Z (larger field), with coordinates x and y; field X was the sagittal slice (smaller field and to the side) with the y and z coordinates; and field Y was the axial slice (smaller field and below), with coordinates x and z.

**Figure 3:**
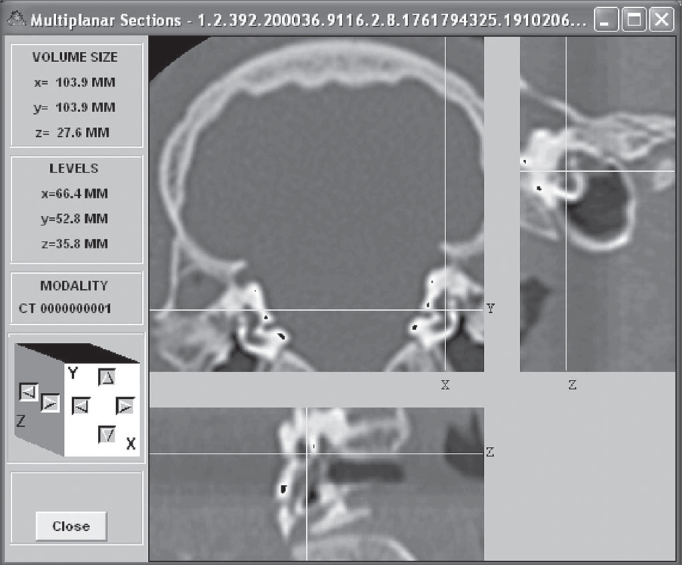
Depict the left ear vestibule height (b1 66.4mm; b2 66.4mm; c1 49.1mm; c2 52.8mm; length 3.7mm) in the coronal plane (field Z). In FS the cursor was placed on the apical face of the vestibule; in FI the cursor was placed on the basal face of the vestibule.

Osiris 4.16 software supplied the coordinates for these points and the distance calculation between the two points was carried out based on the Pythagorean Theorem, which states: any straight line to be measured is the hypotenuse of a triangle rectangle (has a 90° angle), where the hypotenuse is equal to the square root of the sum of the squared catheti. The value of each cathetus is equal to the difference of the values present in the same axis. The use of MS Excel allowed us to calculate the distance from the two coordinates supplied for each measure, by the following equation: a = [(x1 - x2) 2 + (y1 - y2) 2]0.5. This measure is called Euclidian Distance. Through this technique we measured: the width, length and height of the vestibule (Figure 3), length of the modiolus, diameters of the external and internal cochlear basal turn, ray of the internal cochlear basal turn, promontory thickness, diameter and length of the internal acoustic meatus. The length of the cochlear bony canal was calculated by the conic spiral measure, by a competent engineer, considering the modiolus length and the internal diameter of the cochlear basal turn.

### Calculation of the study group and the control group

#### Sample size calculation

Considering α=0.05 and β=0.10 (90% Power), the difference estimate between humans and sheep with size effect (E/S) equal to 1 and a samples ratio of 3:1 (sheep: humans), we calculated a minimum sample size of 36 sheep ears to 12 human ears. Table 1 depicts the estimated error margins for the proposed comparisons.

**Table 1:** Maximum error margins observable to estimate the differences between averages for different inner ear characteristics compared between sheep and humans in samples of 12 and 36 years, respectively.

		Sheep				Human		
Characteristic	average	sd	n	average	sd	n	SE	em
Vestibule width	2,4	0,3	36	2,3	0,55	12	0,13	0,25
Vestibule length	4,1	0,4	36	4,1	1,2	12	0,23	0,46
Vestibule height	3,8	0,4	36	5,6	1,62	12	0,29	0,58
Modiolus length	3,4	0,2	36	4,2	0,73	12	0,13	0,26
External diameter of the cochlear Basal turn	8,2	0,4	36	10,9	0,68	12	0,16	0,32
Internal diameter of the cochlear basal turn	4,9	0,4	36	7,7	0,71	12	0,16	0,33
External diameter of the cochlear medial turn	7,1	0,3	36	8,2	0,65	12	0,14	0,27
Internal diameter of the cochlear medial turn	3,7	0,3	36	6	0,57	12	0,13	0,25
External diameter of the cochlear apical turn	6,2	0,6	36	5,6	0,7	12	0,21	0,42
Internal diameter of the cochlear apical turn	3,7	0,2	36	3,4	0,7	12	0,13	0,26
Ray of the canal at the cochlear basal turn	1,3	0,2	36	1,4	0,19	12	0,07	0,13
Promontory thickness	1,4	0,1	36	1,2	0,23	12	0,05	0,09
Extension of the cochlear bony canal	19,9	1,7	36	31	2,85	12	0,68	1,36
Diameter of the internal auditory meatus	1,6	0,3	36	5	0,96	12	0,18	0,36
Length of the internal auditory meatus	2,1	0,1	36	11,9	0,91	12	0,15	0,30

The measures are presented in millimeters. sd: standard deviation, n: size of the group, se: standard error, em: error margin.

#### Control group characteristics

Adult persons who underwent CT scan by medical request and without any other inner ear disease.

#### Statistical analysis

The data were described according to Tukey, presenting the five basic summaries of a series: minimum, 25th percentile, 50th percentile (median), 75th percentile and maximum. Moreover, both the average and standard deviation were calculated. Following that, we inserted the reference point to the 2/3 of the average obtained for humans in the distribution of values observed for sheep structures. With that, it was possible to obtain the estimated ratio for sheep which had inner ear structures equal or greater than 2/3 of what had been observed for humans.

## RESULTS

Table 2 depicts the results found in the present study.

**Table 2:** Measures of inner ear structures in millimeters comparing sheep and human ears.

Structure	n		sd	2/3	min.	P25	md	P75	max.	
Sheep										

Vestibule width	37	2,4	0,3		1,8	•2,1	2,4	2,6	2,8	
Vestibule length	30	4,1	0,5		3,3	3,8	4,2	4,4	•4,7	
Vestibule height	36	3,8	0,4		2,9	•3,6	3,9	4,1	4,6	
Modiolus length	34	3,4	0,2		3,1	3,3	3,3	3,5	3,8	•
Cochlea ext.bas turn D	38	8,2	0,4		•7,3	7,8	8,3	8,4	8,8	
Cochlea int.bas turn D	37	4,9	0,4		4,0	•4,4	4,9	5,3	5,8	
Cochlea ext.med turn D	37	7,1	0,3		•6,5	6,8	7,1	7,3	7,9	
Cochlea int.med. turn D	36	3,7	0,3		•3,2	3,5	3,7	3,8	4,2	
Cochlea ext.api. turn D	36	6,2	0,6		•5,2	5,7	6,2	6,7	7,6	
Cochlea int. api. turn D	36	3,0	0,2		•2,5	2,8	3,0	3,1	3,4	
Cochlea ray ang bas.	36	1,3	0,2		1,0	1,1	1,3	•1,6	1,8	
Promontory thickness	37	1,4	0,1		•1,2	1,3	1,4	1,4	1,6	
Cochlea can ext.	34	19,9	1,7		16,5	•18,3	20,0	21,3	23,6	
IAM Diameter	38	1,6	0,3		1,1	1,3	1,6	1,8	2,3	•
IAM Length	37	2,0	0,1		1,7	2,0	2,1	2,1	2,2	•

Human										

Vestibule width	14	2,9	0,3	1,9	2,1	2,8	3,0	3,1	3,2	
Vestibule length	14	6,8	0,6	4,5	6,0	6,2	6,6	7,4	7,7	
Vestibule height	14	4,3	0,4	2,9	3,6	4,1	4,3	4,5	5,1	
Modiolus length	14	5,9	0,6	3,9	5,1	5,4	5,7	6,4	6,9	
Cochlea ext.bas turn D	14	9,1	0,7	6,2	8,1	8,5	9,2	9,6	10,1	
Cochlea int.bas turn D	14	6,4	0,7	4,3	5,6	5,9	6,1	7,3	7,7	
Cochlea ext.med turn D	14	6,9	0,6	4,6	5,8	6,4	7,1	7,5	7,7	
Cochlea int.med. turn D	14	4,4	0,4	2,9	4,0	4,0	4,3	4,8	5,3	
Cochlea ext.api. turn D	14	6,2	0,5	4,1	5,2	5,9	6,3	6,5	7,0	
Cochlea int. api. turn D	14	3,5	0,2	2,3	3,1	3,3	3,5	3,7	3,8	
Cochlea ray ang bas	14	2,1	0,1	1,4	1,8	2,0	2,1	2,1	2,3	
Promontory thickness	14	1,3	0,2	0,9	1,0	1,3	1,3	1,5	1,7	
Cochlea can. length	14	26,8	2,8	17,9	23,7	24,7	25,5	30,0	31,5	
IAM Diameter	14	5,2	0,8	3,5	4,1	4,6	5,3	6,0	6,6	
IAM Length	14	13,0	1,2	8,7	10,6	12,3	13,2	14,1	14,7	

n: sample size; : average; sd: standard deviation; min.: minimum; P25: 25th percentile; md: median; P75: 75th percentile; max.: maximum; bas. turnD: diameter of the basal turn; ext.: external; int.: internal; med. turn D: diameter of the medial turn; Api. turn D: diameter of the apical turn; Ray.ang bas.: basal turn canal ray; can length..: canal length; IAM: internal acoustic meatus.

•: represents the positioning of the 2/3 of humans in the series of sheep measures.

## DISCUSSION

The present discussion is limited, considering the unprecedented nature of this paper and the scarce number of publications of this kind. The anatomical description of the sheep ear, despite a careful search in medical and veterinary literature, was found only in investigations carried out by the present author's research group and in textbooks that, frequently, do not mention their references. It should be noted also, that the data is always about sheep and equines, without providing specific references to any of the two species. The only existing publications^1^, ^2^, ^3^, ^4^, ^5^, ^6^, ^7^, ^8^ which resemble this paper have findings which are extremely similar to those from the present study, however aimed at providing a descriptive report of the findings.

Notwithstanding, the present investigation bears original characteristics, because ours is a morphometric and statistical study which came to provide better uniformity to the existing information with a consequent optimization regarding the use of these animals in otologic training and experimental surgeries, such as the investigation on cochlear implants, developing a surgery for endolabyrinthine vertigo or labyrinthectomies, among others^5^.

The anatomical resemblance between sheep and human ears is specially useful for the training and experimentation of procedures such as cochlear implants, considering the lengths of the electrode bundle, which makes possible this type of surgery. Besides these types of surgery, the implantation of hearing aids, transcanal and chemical labyrinthectomies, translabyrinthine neurectomies and saculoutricular surgeries, as well as acoustic trauma studies, may all be trained in sheep, for they have similar measures and a uniform ratio, thus facilitating surgical anatomy understanding. Another advantage is that the surgical accesses are conservative (for instance, there is no need to open the dorsal bone in the sheep head)^5^, ^6^.

Considering that the primary goal of this research was to check and see if the distribution of different inner ear structures sizes of sheep had any relation with their counterparts in human ears, in other words, to compare differences between the groups, the calculation of independent sample average differences was adapted in order to obtain the minimum sample size necessary to carry out the study.

All the sheep exceed the 2/3 of the human average in the following structures: diameter of the cochlear external basal turn; diameter of the cochlear external medial turn, diameter of the cochlear internal medial turn, diameter of the cochlear external apical turn, diameter of the cochlear internal apical turn and promontory thickness. In the 2/3 ratio, 75% of the sheep exceeded humans in the following structures: vestibule width, vestibule height, diameter of the cochlear internal basal turn and extension of the cochlear bony canal. Only the bigger sheep had a 2/3 ratio in the vestibule length. The following structures do not have this 2/3 ratio between sheep and human ears: length of the modiolus, diameter of the internal auditory meatus and length of the internal auditory meatus.

The morphometric study reveals significant similarities between sheep and human ear anatomies. The comparison between sheep ear anatomy, as described in the present paper, and the ear anatomy of other animals, according to what has been described in the aforementioned literature^1^, ^2^, ^3^, ^4^, ^5^, ^6^, ^7^, ^8^, ^9^, ^10^, ^11^, ^12^, ^13^, ^14^, ^15^, ^16^, ^17^, corroborates this concept.

The CT scan measures were made by the author through his prior experience with sheep ear anatomy 8 in each one of the DICOM image sets, obtained from 1mm collimation spiral scans, rebuilt at every 0.5mm, with maximum amplification in order to include both ears in the field of vision. The CT scan device used was a Toshiba, model Xvision EX with simple raceway spiral scans. The data were transferred in the DICOM format to a workstation, where the Osiris 4.16 image editing software was used. In checking the sheep, the number of specimens varied, because some structures were not very clear in the CT scan and the work was based on sustainability and reproducibility measures.

This paper states that based on investigation already carried out on this theme, most of them from the same research group of the present author, we may see that the inner ear histology study mentioned by Lavinsky et al.^5^, shows the same similarity that was seen in this study through macroscopic investigation.

This similarity with human ears establishes a clear statement that sheep are excellent animals for experimental study, and most specially, for ear surgery training. With the current genetic progress in sheep farming, it is possible to create very homogeneous types of animals, thus reinforcing what was said above. The results provide a relevant contribution to this field, considering all the ethical difficulties and, also, the difficulties in having human cadavers to be used in optimizing the training of new otologic surgeons.

Another important point is that the handling of these animals during the different study stages was facilitated by the fact that sheep are docile creatures. There is no need to confine the sheep in a lab. For long observation periods, the sheep may remain in the farms. This reduces cost, besides providing the animals with more comfort and avoiding exposure to potential diseases that the sheep may acquire if they remain in the lab. The care of these sheep in the farm is done by the farmer and does not require the presence o any specialized technician. Thus the low cost and great availability.

Because of its robustness, gentle and docile characteristics, resistance, low maintenance cost, sheep should be used as a special animal model for the training and performance of experimental otologic surgeries^5^.

Therefore, the animals used in these otologic surgeries are free after surgery and may be evaluated for human consumption, three months afterwards. This may reduce or even eliminate costs accruing from the acquisition of these animals. In most of the cases, the sheep are less expensive than dogs (in Brazil a sheep costs about R$75.00, or US$25.00), besides being much more available than the latter^6^, ^7^, ^8^.

## CONCLUSION

From the text we may conclude that:

• There is great similarity between the inner ear anatomy of sheep and humans, according to the measurements done.
• Most of the structures studied (10 to 15) preserved the proposed 2/3 ratio between sheep and human ears.
• There is a great contribution in morphometry for the sheep as an experimental and training model for otologic surgeries.
